# Global Surveillance of Healthcare-Associated Infections in Long-Term Care Facilities: A Narrative Review

**DOI:** 10.3390/microorganisms14061354

**Published:** 2026-06-17

**Authors:** Luisa-Andreea Gheorghe, Carmen-Cristina Vasile, Liviu-Iulian Rotaru, Maria Dorina Crăciun, Irina Magdalena Dumitru, Daniela Pițigoi, Carmen Daniela Chivu

**Affiliations:** 1 Department of Epidemiology I, University of Medicine and Pharmacy ‘Carol Davila’, 020021 Bucharest, Romania; luisa-andreea.ilie@drd.umfcd.ro (L.-A.G.); carmen-cristina.vasile@drd.umfcd.ro (C.-C.V.); maria.craciun@umfcd.ro (M.D.C.); carmen-daniela.chivu@umfcd.ro (C.D.C.); 2 National Administration of Penitentiaries, 023762 Bucharest, Romania; 3 National Institute of Infectious Diseases ‘Prof. Dr. Matei Balș’, 021105 Bucharest, Romania; rotaru.liviu-iulian@s.bio.unibuc.ro; 4 Department of Anatomy, Animal Physiology and Biophysics, Faculty of Biology, University of Bucharest, 050095 Bucharest, Romania; 5 Clinical Emergency Hospital for Children ‘Grigore Alexandrescu’, 011743 Bucharest, Romania; 6 Clinical Infectious Diseases Hospital, 100 Ferdinand Str, 900709 Constanta, Romania; dumitrui@hotmail.com; 7 Faculty of Medicine, Ovidius University of Constanta, Aleea Universității, nr. 1, 900470 Constanta, Romania

**Keywords:** healthcare-associated infections, surveillance, long-term care facilities, review, point prevalence survey, older adults

## Abstract

Healthcare-associated infections (HAIs) in long-term care facilities (LTCFs) are a public health problem worldwide. The aim of this narrative review is to summarize and to provide a comprehensive overview of the current surveillance methods used in LTCFs. We conducted a search of the PubMed database starting from 2000 onward. We found 324 articles that were systematically evaluated by the titles, abstracts, and full texts, and we selected 42 records. These articles were dispersed across North America, Europe, Australia, and Asia. We identified articles describing the results from active or passive surveillance and from a point prevalence study (PPS). The most frequently used method was PPSs in 30 studies, followed by 12 articles reporting active surveillance results and 3 articles describing active surveillance findings. The case definitions applied in 16 articles were those provided by the Centers for Disease Control and Prevention, followed by the McGeer and European Centre for Disease Prevention and Control definitions in 15 and 13, respectively. Twenty-six studies described the method used to calculate the HAI indicator (incidence, incidence density, or prevalence). The articles on PPSs reported prevalence per 100 patients (22 articles), whereas those on active and passive surveillance reported the incidence density per 1000 patient-days. PPSs effectively identified HAIs in LTCFs through standardized data collection and essential interpretive information. The variability in surveillance methods, case definitions, HAI types, and indicator calculation approaches highlights the need for standardized surveillance protocols and methodologies in LTCFs worldwide. Furthermore, these findings emphasize the importance of implementing appropriate surveillance systems and targeted public health measures to effectively address the specific needs and vulnerabilities of the LTCF population in our country.

## 1. Introduction

### 1.1. The Importance of Healthcare-Associated Infections

Healthcare-associated infections (HAIs) are infections that develop in patients during their stay in a hospital or other healthcare facility, such as outpatient clinics, long-term care facilities (LTCFs), rehabilitation settings, and home care, and that were not present or incubating at the time of admission. HAIs can occur in any healthcare setting and may even manifest after the patient’s discharge. The impact of these infections is significantly greater in low- and middle-income countries than in high-income countries (HICs). According to the Global Report on Infection Prevention and Control published by the World Health Organization (WHO) in 2024 on HAIs, an average of 7% of the patients in acute care hospitals in HICs and 15% in low- and middle-income countries (LMICs) are expected to acquire at least one HAI during their hospital stay [1].

HAIs are responsible for extended hospital stays, long-term disabilities, increased antimicrobial resistance, substantial additional costs for healthcare systems, high financial burdens for patients and their families, and preventable deaths. The European Centre for Disease Prevention and Control (ECDC) assessments reveal that the six most frequent HAIs impose a burden twice as substantial as the aggregate burden of 32 other infectious diseases, based on metrics of disability and excess mortality [2].

Despite being the most common adverse event in healthcare, the true global impact of HAIs remains uncertain due to challenges in obtaining reliable data [1].

### 1.2. The Aging Population

Global life expectancy is rising, with most people now expected to live into their sixties or beyond. According to 2019 WHO data, life expectancy was over 70 years (73.1) [3]. Most countries are experiencing increases in both the number and the proportion of older adults. By 2030, one in six people worldwide will be aged 60 or older; as a result, this age group is expected to grow from 1 billion in 2020 to 1.4 billion. By 2050, it will reach 2.1 billion, and those aged 80 or over will triple to 426 million. Although population aging began in HICs, it is now advancing more rapidly in LMICs. By 2050, two-thirds of the global population aged 60 or older will live in these regions [4].

This trend is also clear within the European Union (EU); alongside population aging, the proportion of the very old—those aged 85 and older—is increasing faster than in any other age group. According to Eurostat, life expectancy in Europe in 2024 was estimated to be 81.5 years [5]. Between 2019 and 2050, this segment is expected to more than double, growing from 12.5 million to 26.8 million. The number of centenarians is projected to grow fivefold, reaching nearly 484,000. The rapid growth of the very old population in the EU also poses significant challenges, particularly regarding health and long-term care. As individuals aged 85 and over typically require more intensive support, their growing numbers are expected to place substantial pressure on social services and healthcare systems [6].

In Europe, LTCFs primarily serve elderly populations and include a range of settings, such as nursing homes, residential care homes, and combined facilities.

Romania is facing the same demographic shifts seen across Europe, with a steadily aging population and a declining birth rate, which together are reshaping the country’s age structure. As of recent estimates, over 20% of Romania’s population is aged 65 or older, a proportion projected to increase significantly in the coming decades. By 2050, nearly one in three Romanians is expected to be over 65 years old. Further contributing to this trend is the emigration of working-age individuals, which increases the share of older adults in the population and intensifies the dependency ratio. Without comprehensive policy responses, including investments in age-appropriate healthcare services, long-term care infrastructure, and support for informal caregivers, Romania is likely to face mounting challenges in meeting the needs of its growing elderly population [7,8,9].

### 1.3. HAIs Surveillance

Globally, HAIs are often underreported, with poor data quality and a lack of standardized methods and protocols, resulting in unreliable data, especially in LMICs. Moreover, cultural differences and historical or punitive approaches to documenting HAIs can further influence reporting practices and data accuracy. This data gap, along with the inability to track progress, hampers effective advocacy for infection prevention and control (IPC) resources [2].

### 1.4. Point Prevalence Surveys on HAIs

In Europe, the ECDC monitors the incidence of HAIs and antimicrobial use in medical facilities by conducting periodic point prevalence surveys (PPSs) across EU/EEA (European Economic Area) Member States, following a standardized protocol [10].

Through these PPSs, the ECDC seeks to offer EU/EEA Member States a standardized tool for tracking trends in HAIs and antimicrobial use, to identify key areas for intervention at both national and local levels, to assess the effectiveness of these measures, and to estimate and monitor the impact of HAIs and antimicrobial use [11].

Since 2008, the ECDC has coordinated the surveillance of HAIs and antimicrobial use across Europe, establishing the Healthcare-Associated Infections Surveillance Network (HAI-Net). That same year, the ECDC initiated surveillance of HAIs and antimicrobial use in LTCFs across Europe through the Healthcare-Associated Infections in Long-Term Care Facilities (HALT) project. This initiative incorporated variables from the European Surveillance of Antimicrobial Consumption in Nursing Homes (ESAC-NH) subproject into a protocol for conducting repeated PPSs in LTCFs, providing a comprehensive approach to ongoing evaluation of HAI prevalence, antimicrobial use, and infection prevention and control (IPC) resources across European LTCFs. To date, four HALT surveys have been conducted in Europe: in 2010, 2013–2014, 2016–2017, and 2023–2024 [12,13,14,15].

In the United States of America (USA), the Centers for Disease Control and Prevention (CDC) conducted the first PPS on HAIs in a hospital setting in the 1970s. The findings highlighted the importance of creating a surveillance system to monitor the HAI rates. In 1989, based on the observation that the incidence of HAIs in LTCFs was similar to that in hospitals, the Health Care Financing Administration (now known as the Center for Medicare and Medicaid Services) required LTCFs to develop and maintain infection prevention and control programs [16].

The Pan American Health Organization (PAHO) started a 4-year project in 2020, called ‘Working together to fight antimicrobial resistance’, through which it helped countries across South America conduct PPSs in their territories using the One Health approach. These PPSs focused mainly on antimicrobial consumption [17].

The Global-PPS is a program that provides a standardized data collection protocol tailored to the needs of the medical facility. Data collection forms are available for both inpatient and outpatient settings, with or without the optional HAI module. The basic form includes the ‘type of indication’ for antimicrobial use, categorizing it as HAI (inclusive localization or origin), community-acquired infection, prophylaxis, or unknown. Furthermore, the optional HAI module requires supplementary patient data, such as age, gender, comorbidities, presence of indwelling devices, and McCabe score [18].

PPSs have been implemented globally, ranging from LTCFs to acute care hospitals. Apart from Europe and the USA, China was the first country to conduct a PPS in 2007, while countries such as Japan, Brazil, and Ghana hosted their first PPSs in 2014, 2017, and 2019, respectively [19,20,21,22].

Unlike PPSs, there is no universally standardized protocol for passive and active surveillance in LTCFs. Instead, each country develops its own approach tailored to the specific needs and conditions of its population.

### 1.5. The Aim of the Study

This study aims to synthesize the information and to provide a comprehensive overview of the knowledge and different HAIs surveillance systems used in LTCFs worldwide. Additionally, it seeks to address this gap on HAIs in Romanian LTCFs and to highlight the absence of comprehensive regulatory frameworks in this field, using insights from global surveillance data.

## 2. Materials and Methods

### 2.1. Database and Search Items

We conducted a narrative review by searching the PubMed database for relevant publications in November 2024. The searched items included (“nosocomial infection*” OR “healthcare acquired infection*” OR “health care associated infection*” OR “health care-associated infection*” OR “hospital acquired infection*” OR “hospital-acquired infection*” OR “healthcare associated infection*” OR “HCAI*”) AND (“surveillance” OR “survey*”) AND (“long term care facility” OR “long-term care facility*” OR “geriatric*” OR “elderly” OR “nursing home*” OR “institutionalized” OR “institutionalised”). Initially, 324 articles were identified and screened based on their titles and abstracts. To enhance the comprehensiveness of the search, the reference lists of all included studies were further examined for additional relevant publications.

### 2.2. Inclusion and Exclusion Criteria

This review included English-language articles published between 2000 and 2024 with accessible full texts. The studies addressing passive and active surveillance, and PPSs, in LTCFs were selected for further analysis in this review.

We included articles starting from 2000 to analyze the trend of the current century. Although the articles were published from 2000 onward, three of them reported data from the last decade of the 20th century. We excluded articles that referred to hospital surveillance and pediatric centers. No restrictions were applied based on geographic location.

### 2.3. Study Selection

In the initial stage of our review, we examined 324 articles. Of these, 219 were excluded for reasons such as: being published before 2000 or including terms like “paediatrics,” “children,” or “neonatology” in the title. Two reviewers independently screened each article to determine inclusion. If disagreement arose, a senior reviewer decided. The second stage of the review involved full-text review of the remaining 105 articles to identify those addressing surveillance in LTCFs. An additional 63 articles were excluded during this step, leaving 42 articles for our final analysis.

### 2.4. Data Extraction

One reviewer extracted data from the included studies using a standardized data extraction form. The form included items such as authors’ name, study title, year of publication, case definitions used, inclusion criteria applied to the patients, number of LTCFs and patients, type of surveillance method used, total HAI prevalence or incidence, antibiotic use, and infection types—including bloodstream infection/systemic infection, pneumonia, other lower respiratory tract infection, urinary tract infection, conjunctivitis, gastroenteritis, skin infections and others. Data were collected and organized in a Microsoft Excel database, and the Sankey diagram was generated using the SankeyMATIC web-based visualization tool (https://sankeymatic.com/, accessed on 10 June 2026).

## 3. Results

### 3.1. Type of Surveillance

Out of the forty-two articles, two discussed passive surveillance in LTCFs, ten described active surveillance, twenty-nine presented PPS results, one included data from both passive and active surveillance, and one presented both PPS and active surveillance results. These last two articles encompass distinct LTCFs for each surveillance category; consequently, they were incorporated into the aggregate totals for both types. This methodological adjustment ensures comprehensive representation across surveillance frameworks while avoiding double-counting of overlapping facilities. None of the initial articles were reviews.

### 3.2. Geographical Distribution

**Figure 1:**
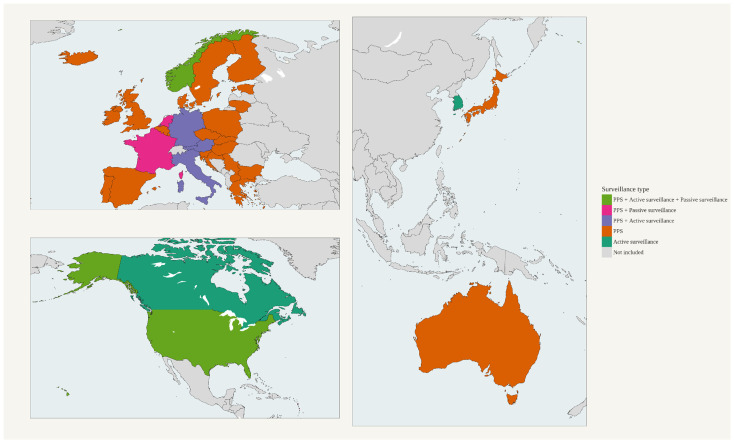
Geographical distribution of the articles by the type of surveillance.

The final set of 42 articles from a diverse range of countries reflects a broad international interest in the subject matter, as shown in Figure 1. Most of the studies (n = 31) were conducted in European countries, including six each from Italy, The Netherlands and Norway, four from Germany and three from Ireland. Seven studies were based in the USA, while the remaining four were conducted in Australia, Canada, Republic of Korea, and Japan, one study from each country. This geographical distribution highlights the global scope of research on the topic and underscores its significance for Romania as a valuable reference, especially considering we did not find any articles from our country on this subject.

### 3.3. Case Definition of HAIs

Case definitions for HAIs varied across the included studies, as presented in Table 1. Clinical and laboratory criteria differed substantially between definitions due to the diversity of HAIs analyzed. Therefore, exhaustive reporting of all individual criteria was not undertaken. Twenty articles used a single source for their case definitions, whereas the others combined and adapted two or three sources. CDC definitions [15,16,23,24,25,26,27,28,29,30,31,32,33,34,35,36] for HAIs were used in 16 studies, McGeer criteria [14,24,29,32,33,34,35,36,37,38,39,40,41,42,43] and ECDC’s [12,13,14,15,23,32,34,44,45,46,47,48,49,50,51] in 15 articles each, and national case definitions in 10 studies [12,13,25,26,27,52,53,54,55,56]. Less frequently used case definitions were those provided by the Society of Healthcare Epidemiology of America (SHEA; 5 studies) [15,28,32,34,53], the Association of Professionals in Infection Control and Epidemiology (APIC; 4 studies) [52,53,57,58] and the Long-Term Care Special Interest Group (LTCSIG; one study) [15]. Two studies did not specify which case definition was used to classify infections as HAIs [59,60].

### 3.4. Passive Surveillance

Three articles addressed passive surveillance of HAIs [23,42,59]. These studies were conducted in Europe (two articles), originating in Norway and France, and in the USA (one article).

These studies were initiated during distinct time periods: 2001, 2006 and 2021.

In aggregate, the three studies implemented passive surveillance across 475 LTCFs, ranging from 9 to 450.

Of the three articles examined, one did not specify the case definitions for HAIs. Among the remaining two, one used criteria from the ECDC and the CDC, and the other used the McGeer case definitions.

All three articles explicitly stated that they included all patients from the LTCFs.

Two studies each delineated the characteristics of the LTCFs, the antimicrobial use within the analyzed population, and the presence of indwelling devices.

Regarding the types of HAIs analyzed in the reviewed articles, gastroenteritis was reported in all three articles, while bloodstream infections (BSIs), urinary tract infections (UTIs), pneumonia, lower respiratory tract infections (excluding pneumonia), skin infections, gastroenteritis, and other rare infections were each described in two articles. In contrast, conjunctivitis was described in only one article.

### 3.5. Active Surveillance

Twelve studies described the results from active surveillance [24,36,40,41,42,43,44,52,54,55,56,58]. Seven of these studies originated in Europe (Norway, Italy, Germany, Austria, The Netherlands), three in the USA, and one each in the Republic of Korea and Canada.

Regarding the starting period of the studies, two articles each were initiated in 2001, 2003, 2004, and 2019, and one article each in 1994, 1998, 2009, and 2018. Between 2006 and 2019, no articles were published on the active surveillance of HAIs in LTCFs.

The total number of LTCFs included across these 11 articles was 95, with a range of 1 to 58 units.

All 12 articles explicitly specified their case definitions. The McGeer criteria and the national and regional protocols were the predominant frameworks, used in five studies each, followed by the definitions from APIC, NHSN, CDC, and ECDC in one article each.

Regarding the inclusion criteria for the patients, 11 studies included all the patients present in the unit at the time of the study. One article, a case–control study, used active surveillance data to measure the risk ratio of complications in patients with a HAI [43]. Six articles each presented data on antimicrobial use, LTCF characteristics (patients’ profiles, room types, bed counts, funding types, etc.), and the presence of any indwelling devices.

The types of HAIs varied across the reviewed articles. UTIs and respiratory tract infections excluding pneumonia were most frequently reported, appearing in ten articles each, followed by pneumonia and gastroenteritis, each documented in nine articles. Skin and other rare infections were presented in eight studies, conjunctivitis was noted in five articles, and BSIs were discussed in three articles.

### 3.6. Point Prevalence Study

A total of 30 articles that used a PPS design were evaluated [12,13,14,15,16,25,26,27,28,29,30,31,32,33,34,35,37,38,39,45,46,47,48,49,50,51,52,53,57,60]. Of these, 27 articles presented data on the prevalence of HAIs, and three articles pursued other objectives.

These studies were conducted globally: four studies each in The Netherlands, Italy, and the USA, three in Ireland, two each in Norway and Germany, and one each in Australia, Scotland, France, Poland, Belgium, and Japan. Four studies included results from PPSs developed by ECDC in multiple European countries as part of the HALT program.

The analyzed articles included 44 distinct PPSs conducted from 1996 to 2024, which evaluated 481,624 residents over different periods. Five articles presented data from the European PPSs in the HALT program and added specific objectives to the studies [28,34,45,50,51].

**Figure 2:**
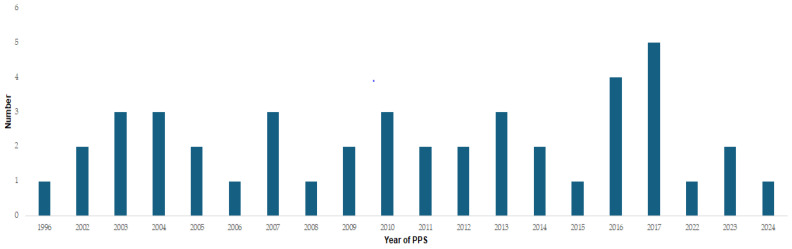
Temporal distribution of PPSs according to the year conducted (N = 44).

The peak year for PPSs was 2017, with five studies, followed by four PPSs in 2016, and three PPSs each in 2003, 2004, 2007, 2010, and 2013, as shown in Figure 2.

All 30 articles provided data on a total of 8858 LTCFs, with each study ranging from 7 to 2221 LTCFs. Most of the studies (28/30) described the data collection methodology, either digital or on paper. Information on the inclusion criteria for patients and HAIs was available from 28 articles. All such studies included every patient present in LTCFs on the survey date, as well as all HAIs exhibiting ongoing clinical manifestations or for which patients were still under treatment at the time of the survey.

Information on the case definition was available in 29 articles. The most frequently employed definitions were those established by the CDC (13 articles), ECDC guidelines (12 articles), McGeer criteria (10 articles), and national protocols (7 articles).

Other published findings in PPSs included antibiotic use, LTCF type, care needs assessment, and risk factors.

**Figure 3:**
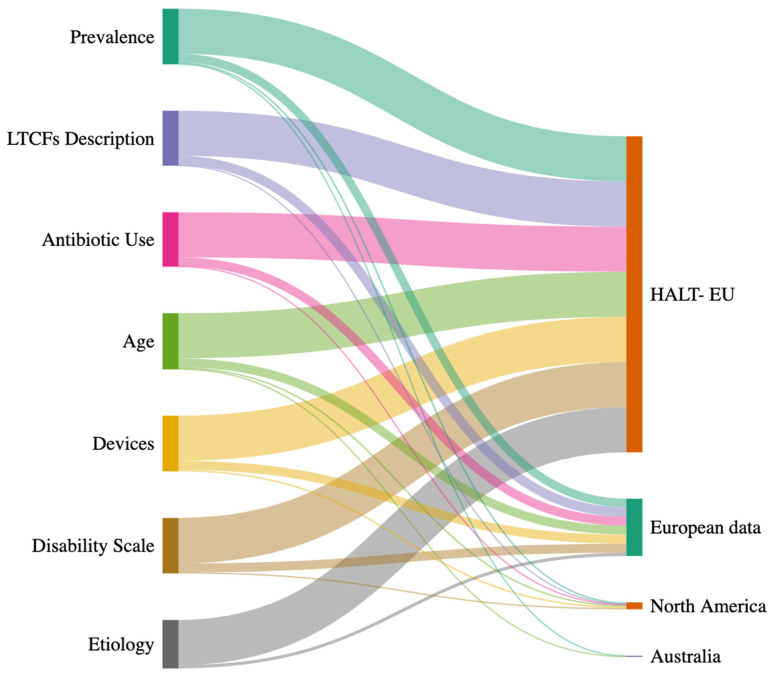
Overview of the published findings in articles regarding PPSs.

Of the 30 articles, 22 presented antibiotic use data, 23 described the LTCF characteristics, and 24 discussed the presence of indwelling devices. These data are presented in Figure 3 and further details are presented in Supplementary Data.

Regarding the distribution of types of HAIs across the analyzed articles, 27 studies reported lower respiratory infections (excluding pneumonia) and UTIs. Pneumonia was surveyed in 24 studies, skin infections in 23, conjunctivitis and gastroenteritis in 19, and BSIs in 18. Additionally, 21 articles reported analyses of other types of HAIs, either specified or unspecified within the respective studies. It was notable that antimicrobial consumption was more frequent in the studies that had higher UTI prevalence and lower in those with higher prevalence for respiratory infections.

Three of the studies were designed as PPSs conducted in LTCFs and did not identify HAIs [35,53,60]. The evaluated aspects included assessing residents’ risk factors, determining the prevalence of colonization with MRSA, ESBL-producing organisms, and *Candida albicans*, and examining the facilities’ capacity and current practices in infection surveillance and control programs.

### 3.7. Indicators

#### 3.7.1. Passive and Active Surveillance

All three studies on passive surveillance described the preferred method for the incidence density of HAIs as follows: one study used the ratio of HAI cases per 10,000 bed-days, while two studies utilized the ratio of HAI cases per 1000 bed-days. Comparing the two studies with similar indicators (incidence density per 1000 patients) revealed differences in their HAI ratios and data collection strategies. The study with the lower incidence value (3.73) used a written form, completed by a nurse, that notified the IPC, whereas the other study used an online form completed weekly by any staff member, capturing all necessary information via an online data collection tool.

Table 2 summarizes the main surveillance approaches used to monitor HAIs in LTCFs, highlighting their methodological characteristics, epidemiological indicators, and practical implications for IPC. PPSs provide prevalence estimates ranging from 2.1% to 11.5% and represent a feasible and cost-effective strategy for obtaining standardized data that facilitate comparisons across facilities and countries. However, because they provide only cross-sectional assessments, they cannot capture temporal variations or rapidly evolving outbreaks. In contrast, active surveillance, based on incidence density measures (0.97–11.8‰), enables continuous monitoring and early outbreak detection, supporting timely and targeted IPC interventions, although it requires substantial personnel and resource investment. Studies based on passive surveillance data relied on routine data collection and were less resource-demanding, but without standardization and variability in implementation. These studies reported incidence densities ranging from 3.73 to 5.37‰.

The key methodological characteristics and IPC implications suggest that selecting a surveillance method requires balancing available resources, surveillance objectives, and the epidemiological context, as each method offers distinct advantages and limitations for monitoring IPC programs in LTCFs.

Regarding active surveillance, nine articles detailed their methods for calculating HAI incidence, with seven studies quantifying incidence density and two calculating incidence rate per 1000 patients, as shown in Table 3. Only two articles reported the number of HAIs; one comparing values before and after hiring IPC personnel without calculating the incidence of HAIs and one case–control study that followed the patients for 30 days to measure reduction in overall physical conditions, transfer to hospital or death [43].

The lowest incidence density (0.97) was reported in a study from the USA that focused exclusively on *Clostridioides difficile* infections in a single LTCF [54]. The highest value (11.8) was reported in a study from Italy that started in 2003. It also reported the highest value for each type of HAI compared with other active surveillance articles; this was also noted by the researchers [58]. A notable difference is that this study was conducted only in summer and autumn. The article references two additional studies from the same country with similar values (7.5 and 12.8). For context, in 2000, Italy’s population was the oldest in the world; it also had a high number of older persons admitted to LTCFs [61].

Eight of the twelve articles were studies conducted throughout the entire year, with values ranging from 0.97 to 6 [36,41,42,44,52,54,55]. These disparities could be attributed to differences in the infection types surveyed, case definitions or local contexts. The other four articles were carried out in different seasons: summer, summer and autumn, winter and spring, and winter, spring and autumn.

Incidence density was higher when external personnel reviewed the charts, likely due to a better understanding of the importance of surveillance.

These findings highlight the importance of continuous surveillance and the role of context in data interpretation.

#### 3.7.2. PPS

Data on prevalence, care load, and risk factors were extracted from 22 articles, as five articles presented findings that were included in larger studies conducted at the European level, and three articles pursued other objectives.

The 44 PPSs conducted from 1996 to 2024 evaluated 481,624 residents with reported prevalence values ranging from 2.1 to 11.5, as shown in Table 3.

The most common infection identified across most studies was UTI, with prevalence rates ranging from 0.8% to 3.93%, followed by respiratory tract infections.

## 4. Discussion

This narrative review aimed to assess knowledge and different surveillance systems used in LTCFs worldwide. It included a variety of studies analyzing various surveillance methods, HAI types, and approaches for calculating HAI prevalence and incidence. The findings also varied by the studies’ geographic locations and time periods.

The review included studies published from 2000 onward, with protocol differences partly due to the varied starting points of the investigations and the broad surveillance timeframe, spanning 1994 to 2023. This chronological diversity mirrors the evolution of surveillance practices over almost three decades in HAI monitoring. Such variation facilitates the analysis of temporal trends in methodological adoption and regional implementation.

Our search method on PubMed yielded no articles regarding LTCFs conducted in Romania, and the country did not participate in any HALT coordinated by ECDC. However, limited data on HAIs in these facilities are available, primarily derived from studies reporting *Clostridioides difficile* infection rates among hospitalized patients originating from such units [62]. Romania has high antimicrobial consumption (28.80 DDD per 1000 inhabitants per day in 2023) and a high prevalence of highly resistant microorganisms (3891 isolates in 2023), thereby increasing the difficulty of managing HAIs [63]. The lack of empirical data on LTCFs in Romania, along with the epidemiological context regarding antimicrobial resistance, highlights the need to address this important knowledge gap.

Most of the reviewed articles presented findings from a PPS, providing the most cohesive and comprehensive dataset. This approach is favored primarily for its logistical feasibility, cost-effectiveness, and alignment with European protocols such as the ECDC’s HALT framework. Given that four European HALTs were included in the analyzed PPS data, we may infer that the most abundant information on the prevalence of HAIs comes from this type of surveillance. Collected data included detailed information regarding the LTCF and the risk factors for each patient (age, mobility, indwelling devices), antimicrobial use (including route of administration and prescribing reasons), and etiological data, including the microorganism involved and its antimicrobial resistance.

In contrast, active surveillance—characterized by ongoing, prospective monitoring—and passive surveillance, which relies on routine reporting, are underrepresented. This is probably attributable to their resource-intensive nature, underreporting biases, and inconsistent implementation outside structured networks. This imbalance underscores a research gap: PPSs are informative for burden estimation but cannot effectively capture variable and evolving trends over time, thereby impeding dynamic policy formulation.

From the initial database, we excluded a study that analyzed infections using the International Classification of Diseases without distinguishing between community-acquired infections and HAIs [64]. We considered this article to not meet the criteria of this review, especially because surveillance that does not differentiate between these two types of infections tends to include minor and mild infections with microorganisms sensitive to antimicrobials, whereas one of the main problems regarding HAIs is that their etiology involves microorganisms with resistance to antimicrobials, and they tend to develop more severely. Additionally, this type of wide surveillance criterion does not help identify an eventual outbreak forming in a LTCF.

The geographical distribution of the included articles reveals a pronounced concentration of research efforts on the surveillance of HAIs within LTCFs in Europe, exceeding that observed in other regions worldwide. This disparity can be attributed to the differences in healthcare infrastructure and surveillance methods. The Eurocentric focus highlights opportunities for international collaboration to harmonize methodologies and mitigate HAIs’ burden on vulnerable elderly populations worldwide, where antimicrobial resistance further complicates outcomes.

Upon examining the case definitions for HAIs selected by providers in the reviewed articles, all included clinical criteria, and most of them included laboratory criteria. Most of the studies used the McGeer and CDC definitions to identify HAIs.

Notably, the McGeer criteria for HAIs place greater emphasis on clinical characteristics than on laboratory evidence, tailored to the operational realities of LTCFs. The ECDC and the national case definitions ranked third and fourth in this distribution. This pattern highlights heterogeneity in the recognition of HAIs within LTCFs. The least-preferred case definitions were those provided by SHEA, APIC, and LTCSIG.

The variability in case definitions suggests that the surveillance objectives were adjusted in response to shifts in population dynamics and the epidemiological context. Although the four European studies commenced in 2010, adapted case definitions were used—including the context of the COVID-19 pandemic, a case criterion was established—that did not produce substantial changes in prevalence estimates, which ranged from 2.1 to 3.7%. The ECDC mentions in the protocol for the third HALT-PPS that their case definitions are developed based on the CDC and SHEA case definitions, and the last two are adapted after McGeer definitions underscoring the importance of the case definitions’ adaptability and flexibility [65].

On the other hand, the highest prevalence (11.5%) was reported in a study from Italy [52], conducted between 2003 and 2006. Later, in 2007 [57], a lower value (8.4%) was reported, and by 2017 [49], the prevalence dropped to 3.9%. These data dynamics suggest interventions rather than variations in the case definition.

However, this observed diversity highlights the critical need for using an actualized standardized case definition of HAIs within the LTCFs. Although there is a discernible trend toward harmonization with the ECDC framework within the EU, substantial progress is still needed to achieve widespread uniformity. Additional use of real-world data allows for more nuanced data and adaptable analyses that better reflect routine clinical practice and diverse healthcare settings.

Significant variability also arises from the specific type of HAIs examined in the included studies. UTIs and lower respiratory tract infections (excluding pneumonia) were the most frequently reported, followed by pneumonia, while BSIs were the least commonly analyzed. These differences in HAI types may reflect epidemiological priorities or data availability in the analyzed studies.

Reporting data on the surveillance of HAIs included the following as indicators: prevalence, incidence, and incidence density. Notably, two articles reported the results as raw counts without deriving a specific indicator.

Data on residents and LTCFs collected and presented in articles based on PPSs are very useful for interpreting study results.

In active and passive surveillance studies, there was heterogeneity in reporting results across different indicator types and limited information on care load and risk factors. However, continuous surveillance compared with PPSs is more useful in identifying outbreaks and tailoring rapid interventions [52].

This variation underscores the importance and influence of surveillance parameters on incidence estimates and reported incidence uniformity. Broad inclusion criteria for patients and infection types can allow us to extract tailored IPC recommendations for each institution.

The interpretation of these data needs to be adapted to the wide diversity of LTCFs, the worldwide spread of the studies, and the particularities of risk factors for each patient type. Regarding the risk factors, we refer to the age of the patients, their comorbidities, any indwelling devices, and their grade of disability. Various methods for evaluating the patients’ disability have been reported, including the case mix index, Katz index, Norton index, Braden scale, cognitive impairment, and activities of daily living, etc. Inconsistencies in the use of disability scales can also affect the comparison between studies because patients with a disability score or with a higher grade of dependency are at higher risk of developing an infection.

The lack of standardization across studies limits the comparability of indicators, thereby affecting the interpretation of interstudy findings. In addition, the heterogeneous reporting of surveillance data further complicates the interpretation and practical use of results. In this context, developing standardized best-practice guidelines for reporting surveillance data, potentially supported by real-world data, could enhance consistency, comparability, and the overall utility of surveillance outcomes.

### 4.1. Strengths

A key strength of the study is its integration of surveillance methods and results from various regions and multiple time periods worldwide. This can be further used to provide regulations for LTCFs, standardized recommendations for surveillance implementation, guidance on the type of HAIs analyzed, and for the uniformity of the indicators. The extensive timeline enables observation of how case definitions, types of surveillance and indicators evolved over time.

When we generated the database, there was no article originating in Romania that discussed the surveillance of HAIs in LTCFs. Therefore, this review could serve as a reference for developing regulations and tailored recommendations for surveillance in LTCFs in our country. Furthermore, there was no review study in the PubMed database.

We included four European PPSs coordinated by ECDC as part of the HALT program, involving 18 to 27 European countries. The ECDC provided standardized case definitions, prevalence calculation methods, data collection forms, and training for the personnel involved in data collection for the PPSs. Romania did not participate in any European PPSs regarding the LTCFs.

### 4.2. Limitations

This study was based on a PubMed-only search strategy, which provided a focused evidence base, but may not capture all publications available in other databases, particularly regional or non-English literature. Additionally, the results retrieved from PubMed could have been influenced by the search strategy, the keyword selection, and the database’s indexing rules which may have affected the identification and inclusion of potentially relevant studies.

## 5. Conclusions

The review identified several critical gaps in active and passive surveillance, the most important being the lack of consistent data across key informational domains. Addressing such gaps through standardized data collection and reporting protocols would strengthen future surveillance efforts in long-term care settings.

PPSs are an important tool for identifying HAIs in LTCFs, by utilizing standardized data collection and providing all the necessary information to interpret the results.

The findings of this study can inform healthcare sector policymakers regarding the critical importance of HAI surveillance in LTCFs, and facilitate the implementation of effective, standardized surveillance programs. These programs would enable targeted control measures to reduce HAI incidence rates, thereby mitigating associated financial burdens and optimizing resource allocation. Such evidence-based strategies align with global priorities for antimicrobial stewardship and infection prevention in resource-constrained settings.

## Figures and Tables

**Table 1:** Criteria included in each HAI definition and frequency of use across studies.

Case Definition	No. of Articles	Clinical Criteria	Laboratory Criteria	Onset of Symptoms	Key Features
McGeer	15	+	-	-	Foundational role in ECDC definitions; emphasis on clinical symptomatology rather than paraclinical evidence; tailored to the operational realities and resource limitations of long-term care facilities (LTCFs)
CDC/NHSN	16	+	+	>3 days after admission	Requires both clinical and laboratory evidence; sets a strict temporal baseline
SHEA	5	+	+	>12 days after admission	Foundational role in ECDC definitions; strict temporal baseline; laboratory evidence
APIC	4	+	+	-	Foundational role in ECDC definitions; strict temporal baseline; laboratory evidence
LTSIG	1	+	+	>12 days after admission	Strict temporal baseline; laboratory evidence; very low use in practice
ECDC 2010	5	+	-	Absent or subclinical	Based on CDC and SHEA guidelines but adapted using McGeer criteria; has flexibility in addressing specific timeframes and emerging threats; serves as the standardized tool for cross-national comparisons in Europe
ECDC 2013	2	+	+-	>48 h after admission <30 days after a surgery One year after a surgery involving an implant	
ECDC 2016	8	+	+	>48 h after admission <30 days after a surgery <90 days after a surgery involving an implant <28 days after discharge from a healthcare facility, CDI	
ECDC 2023	1	+	+	>48 h after admission <14 days for COVID-19	
National case definitions	10	Different criteria depending on the local needs, resources and risks			Highly heterogeneous and dependent on local needs, resources, and risks; limited international comparability

‘+’—criteria present; ‘-’—criteria absent.

**Table 2:** Comparative summary of surveillance approaches in LTCFs.

Surveillance Approach	Primary Indicator (Range)	Key Methodological Characteristics	IPC Implications
PPS	Prevalence (2.1–11.5%)	Logistically feasible and cost-effective; provides a comprehensive dataset aligned with standardized European protocols	Highly useful for global burden estimation and international comparison, but cannot capture dynamic trends over time or track rapid changes
Active Surveillance	Incidence density (0.97–11.8‰)	Resource-intensive, requiring continuous and prospective monitoring; often utilizes external IPC personnel for chart revision	Highly useful for identifying acute outbreaks and allowing facilities to tailor rapid, dynamic interventions
Passive Surveillance	Incidence density (3.73–5.37‰)	Relies entirely on routine reporting by existing facility staff	Susceptible to significant underreporting biases and inconsistent implementation outside of structured networks

**Table 3:** Summary of indicators used in articles regarding PPSs.

Study	Continent, Country	No. PPS	Year of the PPS	Values		Types of HAI	
				No. of Participants	Prevalence Value or Range (%)	Most Common HAI	Prevalence Value or Range (%)
Golliot et al. [29]	EU/EEA, France	1	1996	11,254	9.9	UTI	3.78
Eriksen et al. [27]	EU/EEA, Norway	4	2002–2003	57,769	6.6–7.6	UTI	3.65
Marchi et al. [52]	EU/EEA, Italy	6	2003–2006	4872	8–14	RTI	5.7
Tsan et al. 2008 [16]	North America, USA	1	2005	11,475	5.2	UTI	1.58
Eikelenboom-Boskamp et al. [25]	EU/EEA, The Netherlands	3	2007–2009	4370	7.3	UTI	3.93
Moro et al. [57]	EU/EEA, Italy	1	2007	1926	8.4	RTI	2.8
Tsan et al. 2010 [31]	North America, USA	1	2007	10,939	5.3	UTI	1.64
Mullings et al. [38]	EU/EEA, Scotland	1	2009	922	9.3	UTI	4.9
Eikelenboom-Boskamp et al. 2019 [26]	EU/EEA, The Netherlands	8	2010–2017	9390	1.6–6.7	UTI	0.8–3.5
Latour et al. [14] HALT 1	EU/EEA, 25 countries	2	2010	61,932	2.4	RTI	0.87
Cotter et al. [37]	EU/EEA, Ireland	1	2010	4170	3.7	UTI	1.5
Heudorf et al. [46]	EU/EEA, Germany	1	2011	3732	4.3	UTI	1.2
Willemsen et al. [30]	EU/EEA, The Netherlands	1	2012	774	3.1	UTI	2.33
Latour et al. [15] HALT 2	EU/EEA. 19 countries	1	2013	77,264	3.4	RTI	1.1
Epstein et al. [33]	North America, USA	1	2013	1272	5.3	GII	2
Bennett et al. [39]	Australia	1	2014	3741	2.9	RTI	1.15
Alberg et al. [47]	EU/EEA, Norway	1	2016	23,503	5.2	UTI	2.7
Latour et al. [34] HALT 3	EU/EEA, 24 countries	4	2016–2017	102,301	3.7	RTI	1.25
Furmenti et al. [49]	EU/EEA, Italy	1	2017	24,132	3.9	RTI	1.4
Tandan et al. [32]	EU/EEA, Ireland	1	2017	3816	4.7	UTI	no data
Vicentini et al. [48]	EU/EEA, Italy	1	2022	1025	2.5	RTI	1.27
Aich et al. [12] HALT 4	EU/EEA, 18 countries	3	2023–2024	61,045	3.1	UTI	1.11

UTI—urinary tract infection, RTI—respiratory tract infection, GII—gastrointestinal infection, EU/EEA—European Union/European Economic Area.

## Data Availability

The original contributions presented in this study are included in the article/Supplementary Materials. Further inquiries can be directed to the corresponding author.

## Supplementary Materials

The following supporting information can be downloaded at: https://www.mdpi.com/article/10.3390/microorganisms14061354/s1, Overview of the published findings in articles regarding PPSs.
