# Supplementary material for: Global Surveillance of Healthcare-Associated Infections in Long-Term Care Facilities: A Narrative Review
Source: Microorganisms. 2026 Jun 17;14(6):1354. doi: 10.3390/microorganisms14061354 (PMC13305300; doi:10.3390/microorganisms14061354)
Supplement: Supplementary file 1 [file microorganisms-14-01354-s001.zip › microorganisms-4319494-supplementary.pdf]

Supplementary Material

Overview of the published findings in articles regarding PPSs

Study	Continent, Country	No. PPS	Year of the PPS	Description of the LTCFs	Age	Devices	Disability scale	Prevalence	Antibiotic Use	Ethiology
Golliot et al.	EU/EEA, France	1	1996	+	+	+	+	+	-	-
Eriksen et al.	EU/EEA, Norway	4	2002-2003	+	-	-	-	+	+	-
Marchi et al.	EU/EEA, Italy	6	2003-2006	+	+	+	+	+	-	-
Tsan et al. 2008	North America, USA	1	2005	-	+	+	-	+	-	-
Eikelenboom-Boskamp et al.	EU/EEA, Netherlands	3	2007-2009	+	+	+	+	+	+	-
Moro et al.	EU/EEA, Italy	1	2007	+	+	+	+	+	+	+
Tsan et al. 2010	North America, USA	1	2007	+	+	+	+	+	-	-
Mullings et al.	EU/EEA, Scotland	1	2009	-	+	-	+	+	+	-
Eikelenboom-Boskamp et al. 2019	EU/EEA, Netherlands	8	2010-2017	+	+	+	+	+	+	-
Latour et al. HALT 1	EU/EEA, 25 countries	2	2010	+	+	+	+	+	+	+
Cotter et al.	EU/EEA, Ireland	1	2010	+	+	+	+	+	+	-
Latour et al.*	EU/EEA, Belgium	3	2010, 2013, 2016	+	+	+	+	+	+	-
Heudorf et al.	EU/EEA, Germany	1	2011	+	+	+	+	+	+	-
Eilers et al.*	EU/EEA, Netherlands	1	2012	+	+	+	+	+	+	-

Willemsen et al.	EU/EEA, Netherlands	1	2012	+	+	+	+	+	+	+
Latour et al. HALT 2	EU/EEA. 19 countries	1	2013	+	+	+	+	+	+	+
Roscher et al.*	EU/EEA, Ireland	1	2013	+	+	+	+	-	+	+
Epstein et al.	North America, USA	1	2013	-	+	+	+	+	+	-
Bennett et al.	Australia	1	2014	-	+	-	-	+	-	-
Alberg et al.	EU/EEA, Norway	1	2016	+	+	+	-	+	+	-
Latour et al. HALT 3	EU/EEA, 24 countries	4	2016-2017	+	+	+	+	+	+	+
Suetens et al.*	EU/EEA, 24 countries	4	2016-2017	+	+	+	+	+	+	+
Baranowska-Tetano et al.*	EU/EEA, Poland	1	2017	+	-	+	+	-	+	+
Furmenti et al.	EU/EEA, Italy	1	2017	+	+	+	+	+	+	+
Tandan et al.	EU/EEA, Ireland	1	2017	+	+	+	+	+	+	-
Vicentini et al.	EU/EEA, Italy	1	2022	+	+	+	+	+	+	-
Latour et al. HALT 4	EU/EEA, 18 countries	3	2023-2024	+	+	+	+	+	+	+

*data included in the European PPS-HALT program, EU/EEA - European Union/European Economic Area
